# Three Thousand Consecutive Pancreaticoduodenectomies in a Tertiary Cancer Center: A Retrospective Observational Study

**DOI:** 10.3390/jcm9082558

**Published:** 2020-08-07

**Authors:** Ji Hye Jung, Dong Wook Choi, Sokyung Yoon, So Jeong Yoon, In Woong Han, Jin Seok Heo, Sang Hyun Shin

**Affiliations:** Division of Hepatobiliary-Pancreatic Surgery, Department of Surgery, Samsung Medical Center, Sungkyunkwan University School of Medicine, Seoul 06351, Korea; sog-hei@daum.net (J.H.J.); dwchoi@skku.edu (D.W.C.); sokyung.yoon@samsung.com (S.Y.); sojeong.yoon@samsung.com (S.J.Y.); iw.han@samsung.com (I.W.H.); jinseok.heo@samsung.com (J.S.H.)

**Keywords:** pancreaticoduodenectomy, outcomes, complication, postoperative pancreatic fistula, overall survival

## Abstract

(1) Aim: To evaluate clinicopathological features and postoperative outcomes including survival in patients who underwent pancreaticoduodenectomy (PD) for periampullary diseases. (2) Methods: We retrospectively reviewed 3078 cases of PD performed in our center for 25 years. Periampullary diseases were divided into benign and malignancy groups. All cases were also classified by location. The time of 25 years was divided to different periods (5 years per period) to compare outcomes. Overall survival was compared between subdivided periods. (3) Results: Hospitalization became significantly shorter from 28.0 days in the 1st period to 13.8 days in the 5th period. Overall complication rate was significantly increased since the 3rd period. The rate without postoperative pancreatic fistula (POPF) was high at 98.7% in the 1st period. This might be because drain amylase on the 3rd day after PD was not routinely checked in the past. Thus, POPF was not detected. In survival analysis of adenocarcinoma of pancreas, bile duct, and ampulla, overall survival was found to be improved significantly in recent years. (4) Conclusions: Our study revealed that outcomes were improved with increasing number of PDs performed. Although POPF and overall complications showed increases more recently, those were detected and managed, resulting in shorter hospitalization and improved outcomes.

## 1. Introduction

Pancreaticoduodenectomy (PD) is a surgical treatment of choice for patients with resectable periampullary tumor. PD is one of the most complex and risky procedures. It requires a high level of experience. It was popularized by Whipple and colleagues in the 1930s and 1940s [1,2]. Since then, more and more PDs have been performed worldwide. PD has become a relatively safe operation in many large volume centers. Many surgeons have developed and reported their experiences of PDs and described their outcomes [3,4,5,6,7,8,9,10,11,12]. Representatively, after comparing and studying the existing Whipple operation and the method of preserving the pylorus, it can be said that the popularization of pylorus preserving PD (PPPD) is a very important history [13,14,15,16,17,18].

There have been many efforts and studies to reduce major complications after PD. In terms of postoperative pancreatic fistula (POPF) as a unique complication of pancreatectomy, many studies have been performed in an attempt to identify risk factors and develop surgical techniques or equipment to reduce POPF [19,20,21,22,23]. Other studies have been conducted on delayed gastric emptying (DGE) as a characteristic complication of PD. Antecolic reconstruction has been standardized in gastro- or duodeno-enteric anastomosis currently [24,25,26,27].

In terms of postoperative long-term outcomes, survival rates of patients with periampullary diseases who undergo PD have been gradually improved over time [8,9,11], although the overall survival of patients with pancreatic cancer is still very low [3,4,5,6,7,8,10,12]. These improvements would be the result of complex synergies of various efforts and strategies used by surgeons to improve postoperative outcomes as well as the development of diagnosis and adjuvant therapy by physicians rather than one change in a specific factor.

Therefore, there would be a need to organize outcomes of PD performed at a large institution for a long time. Thus, the purpose of the present study was to analyze clinicopathological features perioperatively and identify chronological changes of postoperative outcomes including overall survival rate in patients who underwent PD for periampullary diseases over the past 25 years at our single tertiary cancer center.

## 2. Materials and Methods

### 2.1. Patients Database

In the present study, we searched consecutive patients who underwent pylorus preserving pancreaticoduodenectomy (PPPD), pylorus resecting pancreaticoduodenectomy (PRPD), hepatopancreaticoduodenectomy (HPD), or total pancreatectomy (TP) performed for periampullary diseases at Samsung Medical Center in Seoul, South Korea, from December 1994 (when our center was opened) to December 2018. Among these, we excluded cases of palliative surgery, cases with double primary cancer, and cases that specific procedure could not be performed due to distant metastasis. In this way, a total of 3078 cases were selected as subjects of this study. Data were collected using electronic medical records of our center and reviewed retrospectively. Information about whether patients were dead or alive and date of death was important; they were all collected from electronic medical records. The department of records in our center collects this information from the national health insurance system. This study was approved by the Institutional Review Board (IRB) of Samsung Medical Center. Our IRB waived the need for written informed consent from participants due to retrospective design of this study.

To identify clinicopathological, surgical, and postoperative characteristics, periampullary diseases were divided into two categories: non-malignancy and malignancy. All cases were also classified based on locations: pancreas, bile duct, ampulla, and duodenum.

Among many factors described, methods for preoperative biliary drainage included percutaneous transhepatic biliary drainage (PTBD), endoscopic retrograde biliary drainage (ERBD), endoscopic naso-biliary drainage (ENBD), metal stent, and others. Whether the pancreatic duct was dilated or not was based on a diameter of 5 mm. Cases of vascular resection were mostly segmental resection and anastomosis of portal vein (PV) or superior mesenteric vein (SMV) because of tumor infiltration or hard adherence to PV or SMV.

Although the staging standard for malignant tumor has been updated several times, we commonly reset the stage of entire cases for 25 years according to the 8th American Joint Committee on Cancer (AJCC) Staging System [28]. Regarding resection margin among pathological features, R0 means both grossly and microscopically margin-negative resection. R1 indicates the removal of all macroscopic tumors while microscopic margins are positive for tumor. R2 means gross residual tumor not resected. In pancreatic cancer, R1 is redefined as a distance of the tumor from the resection margin of ≤1 mm from 2011 [29,30].

Postoperative pancreatic fistula (POPF), a major complication after PD, was evaluated based on the criteria of the International Study Group of Pancreatic Fistula (ISGPF) [31,32]. There are several common major complications besides POPF after PD [33,34]. Overall complications that occurred within 90 days after surgery were graded according to the Clavien–Dindo classification [35]. In survival analysis, we excluded the last two years (2017–2018) to obtain two years of follow-up.

### 2.2. Statistical Analysis

All statistical analyses for comparing clinical, operative, pathologic, and postoperative features and analyzing survival rate were conducted using IBM SPSS statistical software, version 24 (Chicago, IL, USA). Differences with probability (*p*) value 0.05 or less were considered statistically significant. To compare differences among the four groups divided by location, we used one-way analysis of variance, a method for testing differences among more than two groups. Categorical variables were analyzed using a Chi-square test to see differences among the four groups. Overall survival rate was estimated using life table method and survival curves were constructed by Kaplan–Meier survival curves method. Differences in survival were evaluated using the log-rank test.

## 3. Results

### 3.1. Demographic, Clinicopathologic, and Perioperative Characteristics

**Figure 1:**
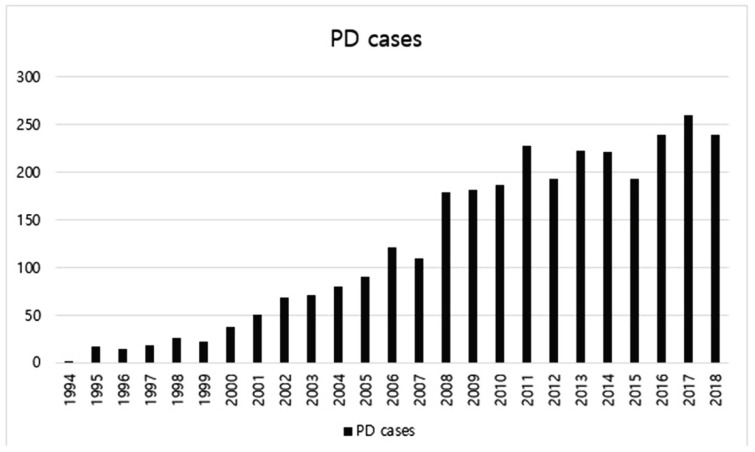
The number of pancreaticoduodenectomy (PD) cases by year from 1994 to 2018.

A total of 3078 PDs were performed in our center for 25 years. The median follow-up period of all enrolled patients was 32 months (range 3–291 months). Figure 1 shows the number of PDs by year. The number was increased generally as years went by. Only 622 cases were performed for the first 13 years whereas 2456 cases were performed for the next 12 years. Regarding locations of diseases receiving PD, the pancreas accounted for the most, followed by bile duct, ampulla, and duodenum. Benign cases accounted for about 15%.

Table 1 shows demographic and perioperative features of 463 benign cases. Incidence of benign disease was higher in men. The average age was 50′s regardless of disease location. The most common benign diseases at pancreas, bile duct, ampulla, and duodenum were pancreatic intraductal papillary mucinous neoplasm (IPMN), biliary inflammation, ampullary adenoma, and duodenal gastrointestinal stromal tumor (GIST), respectively.

Demographic, clinical, and operative features of 2615 patients with malignancy are shown in Table 2. Their average age was 60′s, higher than that of patients with benign diseases. What was common with benign cases was that the incidence was also higher in men and more patients with underlying diabetes were found in pancreatic cases (22.9% in benign and 37.1% in malignancy). Preoperative total bilirubin level was higher in bile duct cases (average 3.3 mg/dL in benign and 8.8 mg/dL in malignancy). Moreover, it was also common that patients with bile duct disease had biliary drainage more often (61.9% in benign and 84.1% in malignancy). The proportion of patients with increased CA 19-9 level was high in those with pancreatic (64.1%) and bile duct malignancy (53.5%). In pancreatic malignancy, the pancreas texture was often hard (72.1%), and vascular resection was frequently performed (24.3%).

Pathologic and postoperative features of malignant cases are shown in Table 3. Adenocarcinoma and moderately differentiation had the highest proportion regardless of its locations. The rate of T2 was high in pancreatic (65.2%) and bile duct cancers (50.3%). T1 and T2 accounted for a large proportion in ampullary cancer (63.6%) whereas T4 was mostly found in duodenal cancer (61.8%). In bile duct cancer, 376 out of 823 cases had T stage unknown. Because T stage of bile duct cancer was defined according to the depth of invaded bile duct wall by the 8th AJCC Staging System, these 376 cases had no information about the depth of invaded wall. More than 20 lymph nodes on average were dissected in all locations. The rate of R0 resection was more than 90% in bile duct, ampullary, and duodenal cancer, whereas it was lower in pancreatic cancer.

The entire time of 25 years was divided into five periods (5 years per period) to compare postoperative outcomes chronologically. Table 4 shows chronologic changes of POPF, length of stay, and overall complication by Clavien–Dindo classification. The rate of no POPF was significantly high in the 2nd period (98.7%) compared to that in the 3rd period (90.9%), 4th period (89.4%), and the 5th period (91.8%) (*p* = 0.001, *p* < 0.001, and *p* = 0.001 respectively). The rate of no POPF was about 90% since the 3rd period. The length of stay was significantly decreased from 28.0 days in the 1st period to 13.8 days in the 5th period (*p* < 0.001). Overall complication rate was significantly increased since the 3rd period compared to that in the 1st period (*p* = 0.002) and the 2nd period (*p* < 0.001).

In the past, there were many cases that were not recorded at all. Thus, there were several factors that have many missing data. Fortunately, there were relatively few missing data in important factors such as operation type, pathology, tumor size, and others.

### 3.2. Survival Outcomes

**Figure 2:**
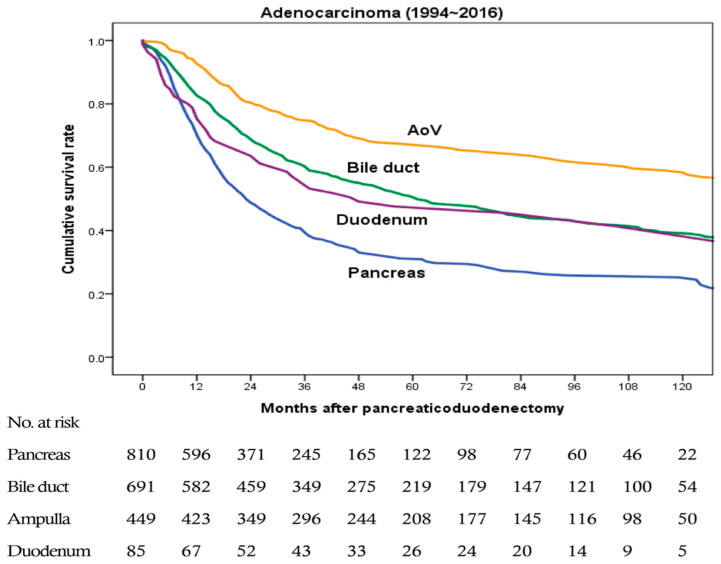
Overall survival rates of patients with pancreatic, bile duct, ampullary, and duodenal adenocarcinomas from 1994 to 2016 (a total 2035 cases, 810, 691, 449, and 85 cases, respectively). Pancreatic vs. bile duct, *p* < 0.001; pancreatic vs. ampullary, *p* < 0.001; pancreatic vs. duodenal, *p* = 0.025; bile duct vs. ampullary, *p* < 0.001; bile duct vs. duodenal, *p* = 0.278; and ampullary vs. duodenal, *p* < 0.001.

Figure 2 shows overall survival rates of patients with pancreatic, bile duct, ampullary, and duodenal adenocarcinomas from 1994 to 2016. The last two years were excluded from survival analysis to obtain two years of follow-up. In malignant cases, survival rate was analyzed only for patients with adenocarcinoma because adenocarcinoma is the most common malignant tumor, and bias is expected to occur when results are analyzed with other histological malignancies. There were a total 2035 cases of adenocarcinoma (810, 691, 449, and 85 cases of pancreatic, bile duct, ampullary, and duodenal adenocarcinomas, respectively). Median survival time were 24, 63, 120, and 50 months, respectively. Overall survival rate was the lowest for those with pancreatic adenocarcinoma and the highest for those with ampullary adenocarcinoma. The difference in survival rate was significant in comparisons of pancreas vs. bile duct, pancreas vs. ampulla, bile duct vs. ampulla, and ampulla vs. duodenum (all *p* < 0.001). It was also significant between pancreas and duodenum (*p* = 0.025). However, the difference in survival rate was not significant between bile duct and duodenal adenocarcinomas (*p* = 0.278).

**Figure 3:**
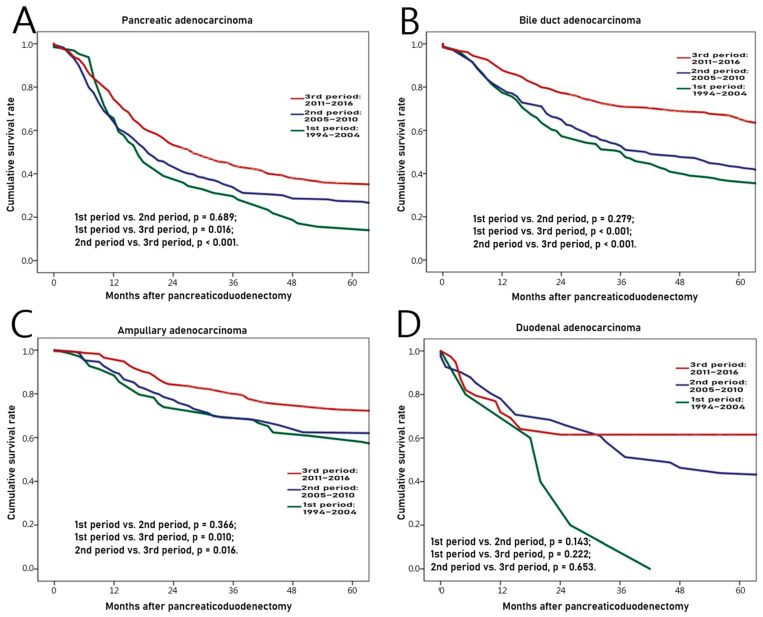
Chronologic differences of survival rates for those with each adenocarcinoma from 1994 to 2016. (**A**) Pancreas, (**B**) Bile duct, (**C**) Ampulla, (**D**) Duodenum.

The overall survival rate was compared between subdivided periods of the 1st (from 1994 to 2004), the 2nd (from 2005 to 2010), and the 3rd (from 2011 to 2016) to find chronologic differences in each adenocarcinoma. As shown in Table 5 and Figure 3A, there were 64, 269, and 477 cases of pancreatic adenocarcinoma in 1st, 2nd, and 3rd periods, respectively. The survival rate was significantly higher as it came to the latest (1st period vs. 2nd period, *p* = 0.689; 1st period vs. 3rd period, *p* = 0.016; and 2nd period vs. 3rd period, *p* < 0.001). As shown in Table 5 and Figure 3B, there were 164, 214, and 313 cases of bile duct adenocarcinomas in 1st, 2nd, and 3rd periods, respectively. The latest survival rate was also significantly higher (1st period vs. 2nd period, *p* = 0.279; 1st period vs. 3rd period, *p* < 0.001; and 2nd period vs. 3rd period, *p* < 0.001). As shown in Table 5 and Figure 3C, there were 69, 149, and 231 cases of ampullary adenocarcinoma in 1st, 2nd, and 3rd periods, respectively. Likewise, the latest survival rate was significantly improved (1st period vs. 2nd period, *p* = 0.366; 1st period vs. 3rd period, *p* = 0.010; and 2nd period vs. 3rd period, *p* = 0.016). For those with pancreatic, bile duct, and ampullary adenocarcinomas, the survival rate was significantly improved as time went by. For those with duodenal adenocarcinoma, recent survival rate was also higher. However, the difference in survival rate by period was not significant as shown in Table 5 and Figure 3D (1st period vs. 2nd period, *p* = 0.143; 1st period vs. 3rd period, *p* = 0.222; and 2nd period vs. 3rd period, *p* = 0.653). There were 5, 41, and 39 cases of duodenal adenocarcinoma in 1st, 2nd, and 3rd periods, respectively.

## 4. Discussion

PD is a necessary surgical treatment for patients with resectable periampullary tumors. Since PD was first performed, the number of PDs has been increasing, and surgical outcomes including overall survival have been improved. Outcomes of PDs have been reported in many studies for a long time [3,4,5,6,7,8,9,10,11,12]. Our study showed similar results. From 1994 to 2018 (for 25 years), the frequency of PD performed was generally increasing year by year. The length of stay in hospital became significantly shorter as time went by. The overall survival rate was also significantly improved.

In our center, general surgeons performed all operations for organs of abdomen, while specialist surgeons in biliary and pancreatic area have been in charge of PD since 2005. Since then, the number of PDs has increased year by year. The standard practice of management called “critical pathway (CP)” began in 2013 based on abundant clinical experiences of specialists. Advances in surgical techniques, equipment, and intensive perioperative care system as well as large volume institution all contributed to the stability of PD and improvement of postoperative outcomes.

Although POPF occurred significantly less in the first two periods than later, this was because of the fact that drainage amylase was not routinely measured at that time. Thus, those cases were considered to have no POPF. Since the third period, it was a routine practice to measure drainage amylase level on the third day after PD. Thus, the occurrence of POPF could be detected well without being missed. Therefore, appropriate management could be done and hospitalizations could not be prolonged. Even after drain amylase was routinely measured, the rate of no POPF cases was maintained at around 90%.

Another point to discuss in our study is the rate of overall complications. Although overall complications according to the Clavien–Dindo classification might appear to be significantly increased since the third period (since 2004), it was thought that more activation of blood transfusion, total parenteral nutrition, and intravenous antibiotics might have increased the rate of cases with grade II complications. The increase in grade IIIa complications might be due to further development of endoscopic and radiologic interventions such as percutaneous catheter drainage, biliary drainage, angiography, vessel embolization, and others.

When the overall survival rate of patients with adenocarcinoma was compared by location, the survival rate of those with pancreatic adenocarcinoma was significantly lower than that of patients with adenocarcinoma at other locations in our study, consistent with many previous reports [3,4,5,6,7,8,10,12]. The survival rate of those with ampullary adenocarcinoma was the highest in our study. However, some studies have reported that those with duodenal adenocarcinoma have the highest survival rate [3,4,6]. Such discrepancy might be because duodenal adenocarcinoma has a lower incidence in our center. In addition, those with duodenal adenocarcinoma are often slower to show symptoms. Thus, cases were found at a later stage. Actually, this study showed that the rate of T4 in duodenal malignancy was high.

We discussed several postoperative outcomes in this study. The most important finding of this study was that the overall survival of patients with periampullary malignancy was improved. Above all, resectability and complete resection including mesopancreas excision would be the most significant contributing factors [36,37,38,39,40], and help of adjuvant chemotherapy would be added [41,42,43,44]. If tumor can be diagnosed early, resectability can be increased [45], so many studies have been performed to find related genes [46,47,48]. In addition, detailed strategies during PD have been studied to advance surgical outcomes, including division of surgeon workload, whether to put the pancreatic stent externally or internally, the relationship between the amount of intraoperative fluid during PD and POPF, and others [49,50,51].

This study has several limitations. First, information on death and date of death was entirely based on electronic medical records of our center. There might be missing information that the department of records did not collect. Our country has a national health insurance system which covers the whole population. If anyone dies, information about the fact and date of death is reported to the government department. The department of records in our center periodically collects such information from the government departments and saves it in electronic medical records. That is not a real-time collection but a periodic collection, so there may be information that has been missed due to a long interval of input period. Second, as a retrospective observational study, accurate and detailed data could not be obtained from old records. For example, in the past, the length of hospitalization after PD was long. However, there was little record of why it was long, making it impossible to know the accurate progression. In addition, tests that had to be done after PD were not routine. To overcome this limitation, we established a web-based database to collect all data for patients undergoing hepato-bilio-pancreatic surgeries including PD since 2017, and postoperative essential evaluations are routinely performed following the “CP”.

Despite these limitations, through this descriptive study, we could analyze various factors of 3078 cases of PDs performed over the past 25 years and identify improved surgical outcomes including overall survival. The overall survival rate was analyzed by dividing the entire period into three periods after dividing cases according to the location of adenocarcinoma so that we could identify more specific results about overall survival. Chronological analyses of the incidence of POPF, hospitalization days, and overall complication by dividing the entire 25 years into five periods also provided more information.

## 5. Conclusions

As time went by, PD was increasingly performed for patients with periampullary diseases, and surgical outcomes were improved. Our study identified an increasing number of PDs for 25 years with significantly improved outcomes, including shorter hospital stays and improved overall survival. Although POPF and overall complications were found to have increased in recent years, those complications were detected without being missed and cases could be managed effectively, resulting in shorter hospitalization and improved outcomes. Thus, advanced stability of PD and improved postoperative outcomes were confirmed in our study. It is obvious that PD has contributed to the treatment of periampullary diseases.

## Figures and Tables

**Table 1:** Clinical, operative, pathologic, and postoperative features of 463 patients with non-malignant disease. (Mean ± Standard deviation and number (percent)).

	Pancreas (*n* = 362)		Bile Duct (*n* = 21)		Ampulla (*n* = 44)		Duodenum (*n* = 36)	
Age	57.7 ± 12.6		57.1 ± 10.2		60.1 ± 10.5		54.3 ± 13.1	
Sex								
Male	216 (59.7)		16 (76.2)		28 (63.6)		20 (55.6)	
Female	146 (40.3)		5 (23.8)		16 (36.4)		16 (44.4)	
Body weight (kg)	62.6 ± 10.5		64.2 ± 10.6		65.5 ± 12.8		65.1 ± 7.9	
BMI (kg/m^2^)	23.5 ± 3.3		23.5 ± 3.7		24.5 ± 4.3		24.4 ± 2.7	
DM								
No	279 (77.1)		17 (81.0)		39 (88.6)		32 (88.9)	
Yes	83 (22.9)		4 (19.0)		5 (11.4)		4 (11.1)	
ASA score								
1	101 (27.9)		6 (28.6)		11 (25.0)		16 (44.4)	
2	242 (66.9)		14 (66.6)		31 (70.5)		20 (55.6)	
3	16 (4.4)		1 (4.8)		2 (4.5)		0 (0.0)	
Unknown	3 (0.8)		0 (0.0)		0 (0.0)		0 (0.0)	
Preoperative total bilirubin (mg/dL)	1.2 ± 2.2		3.3 ± 3.3		1.0 ± 1.3		0.7 ± 0.6	
Preoperative biliary drainage								
No	333 (92.0)		8 (38.1)		28 (63.6)		34 (94.4)	
Yes	29 (8.0)		13 (61.9)		16 (36.4)		2 (5.6)	
Operation type								
PPPD	267 (73.8)		17 (81.0)		38 (86.4)		23 (63.9)	
PRPD	62 (17.1)		4 (19.0)		5 (11.4)		13 (36.1)	
HPD	0 (0.0)		0 (0.0)		1 (2.2)		0 (0.0)	
TP	33 (9.1)		0 (0.0)		0 (0.0)		0 (0.0)	
Pancreas texture								
Soft	147 (40.6)		9 (42.9)		25 (56.8)		20 (55.6)	
Hard	134 (37.0)		8 (38.1)		12 (27.3)		12 (33.3)	
Unknown	81 (22.4)		4 (19.0)		7 (15.9)		4 (11.1)	
Pancreatic duct								
≤5 mm	236 (65.2)		17 (81.0)		36 (81.8)		33 (91.7)	
>5 mm	59 (16.3)		0 (0.0)		1 (2.3)		0 (0.0)	
Unknown	67 (18.5)		4 (19.0)		7 (15.9)		3 (8.3)	
Pancreas reconstruction								
PJ	326 (90.0)		21 (100.0)		43 (97.7)		36 (100.0)	
PG	2 (0.6)		0 (0.0)		1 (2.3)		0 (0.0)	
None	34 (9.4)		0 (0.0)		0 (0.0)		0 (0.0)	
Vascular resection								
No	352 (97.2)		21 (100.0)		44 (100.0)		36 (100.0)	
Yes	10 (2.8)		0 (0.0)		0 (0.0)		0 (0.0)	
Combined operation								
No	324 (89.5)		20 (95.2)		42 (95.5)		35 (97.2)	
Yes	38 (10.5)		1 (4.8)		2 (4.5)		1 (2.8)	
Operation duration (minutes)	307.6 ± 78.3		343.1 ± 86.4		322.1 ± 92.2		291.1 ± 68.1	
EBL (mL)	489.5 ± 638.4		554.8 ± 317.8		396.5 ± 264.2		447.2 ± 371.3	
Intraoperative transfusion								
No	321 (88.7)		17 (81.0)		40 (90.9)		31 (86.1)	
Yes	41 (11.3)		4 (19.0)		4 (9.1)		5 (13.9)	
Pathology	IPMN	194 (53.6)	Inflammation	8 (38.1)	Adenoma	37 (84.1)	GIST	27 (75.0)
	NET	50 (13.8)	Adenoma	7 (33.3)	NET	4 (9.1)	Adenoma	5 (13.9)
	Inflammation	41 (11.3)	Cyst	5 (23.8)	Carcinoid	2 (4.5)	NET	3 (8.3)
	SCN	29 (8.0)	Neuroma	1 (4.8)	GIST	1 (2.3)	Leiomyoma	1 (2.8)
	SPN	27 (7.5)						
	MCN	13 (3.6)						
	Etc.	8 (2.2)						
Tumor size (cm)	3.6 ± 2.4		1.9 ± 1.3		1.9 ± 1.3		5.0 ± 3.0	
Length of stay (days)	15.2 ± 9.8		16.7 ± 10.4		14.8 ± 11.6		13.1 ± 5.1	
POPF								
No (Biochemical leak)	298 (90.6)		19 (90.5)		42 (95.5)		30 (83.3)	
Grade B	29 (8.8)		2 (9.5)		2 (4.5)		6 (16.7)	
Grade C	2 (0.6)		0 (0.0)		0 (0.0)		0 (0.0)	
TP	33		0		0		0	
Clavien–Dindo classification								
No complication	233 (64.4)		16 (76.1)		32 (72.8)		21 (58.3)	
I	16 (4.4)		1 (4.8)		2 (4.5)		4 (11.1)	
II	60 (16.6)		2 (9.5)		5 (11.4)		8 (22.3)	
IIIa	36 (9.9)		1 (4.8)		3 (6.8)		3 (8.3)	
IIIb	7 (1.9)		1 (4.8)		2 (4.5)		0 (0.0)	
IVa	7 (1.9)		0 (0.0)		0 (0.0)		0 (0.0)	
IVb	2 (0.6)		0 (0.0)		0 (0.0)		0 (0.0)	
V	1 (0.3)		0 (0.0)		0 (0.0)		0 (0.0)	

BMI: body mass index, DM: diabetes mellitus, ASA: American Society of Anesthesiologists, PPPD: pylorus preserving pancreaticoduodenectomy, PRPD: pylorus resecting pancreaticoduodenectomy, HPD: hepatopancreaticoduodenectomy, TP: total pancreatectomy, PJ: pancreaticojejunostomy, PG: pancreaticogastrostomy, EBL: estimated blood loss, IPMN: intraductal papillary mucinous neoplasm, NET: neuroendocrine tumor, SCN: serous cystic neoplasm, SPN: solid pseudopapillary neoplasm, MCN: mucinous cystic neoplasm, GIST: gastrointestinal stromal tumor, POPF: postoperative pancreatic fistula. Combined operation: splenectomy (15 cases), colectomy (4 cases), nephrectomy (4 cases), appendectomy (4 cases), and others.

**Table 2:** Clinical and operative features of 2615 patients with malignant tumor (Mean ± Standard deviation and number (percent)).

	Pancreas (*n* = 1096)	Bile Duct (*n* = 823)	Ampulla (*n* = 565)	Duodenum (*n* = 131)
Age	62.3 ± 10.5	64.3 ± 9.2	62.1 ± 10.5	61.2 ± 10.8
Sex				
Male	660 (60.2)	535 (65.0)	318 (56.3)	88 (67.2)
Female	436 (39.8)	288 (35.0)	247 (43.7)	43 (32.8)
Body weight (kg)	60.1 ± 10.1	61.0 ± 9.7	61.6 ± 10.5	63.0 ± 12.0
BMI (kg/m^2^)	22.8 ± 3.0	23.2 ± 3.0	23.5 ± 3.3	23.6 ± 3.7
DM				
No	689 (62.9)	663 (80.6)	463 (81.9)	110 (84.0)
Yes	407 (37.1)	160 (19.4)	102 (18.1)	21 (16.0)
ASA score				
1	205 (18.7)	170 (20.7)	131 (23.2)	37 (28.2)
2	794 (72.4)	568 (69.0)	394 (69.7)	82 (62.6)
3	92 (8.4)	78 (9.5)	39 (6.9)	11 (8.4)
4	3 (0.3)	1 (0.1)	0 (0.0)	1 (0.8)
Unknown	2 (0.2)	6 (0.7)	1 (0.2)	0 (0.0)
Preoperative total bilirubin (mg/dL)	5.9 ± 7.2	8.8 ± 8.6	4.8 ± 6.5	2.2 ± 4.2
Preoperative CEA				
Normal (≤5 ng/mL)	822 (75.0)	644 (78.3)	449 (79.5)	97 (74.0)
Elevated (>5 ng/mL)	116 (10.6)	44 (5.3)	20 (3.5)	15 (11.5)
Unknown	158 (14.4)	135 (16.4)	96 (17.0)	19(14.5)
Preoperative CA19-9				
Normal (≤37 U/mL)	368 (33.6)	359 (43.6)	345 (61.1)	82 (62.6)
Elevated (>37 U/mL)	703 (64.1)	440 (53.5)	198 (35.0)	36 (27.5)
Unknown	25 (2.3)	24 (2.9)	22 (3.9)	13 (9.9)
Preoperative biliary drainage				
No	469 (42.8)	131 (15.9)	224 (39.6)	115 (87.8)
Yes	627 (57.2)	692 (84.1)	341 (60.4)	16 (12.2)
Operation type				
PPPD	568 (51.8)	533 (64.8)	472 (83.6)	35 (26.7)
PRPD	398 (36.3)	234 (28.4)	90 (15.9)	91 (69.5)
HPD	3 (0.3)	46 (5.6)	0 (0.0)	3 (2.3)
TP	127 (11.6)	10 (1.2)	3 (0.5)	2 (1.5)
Pancreas texture				
Soft	187 (17.1)	341 (41.4)	266 (47.1)	64 (48.9)
Hard	790 (72.1)	281 (34.2)	214 (37.9)	46 (35.1)
Unknown	119 (10.8)	201 (24.4)	85 (15.0)	21 (16.0)
Pancreatic duct				
≤5 mm	770 (70.3)	626 (76.0)	437 (77.4)	103 (78.6)
>5 mm	183 (16.7)	22 (2.7)	56 (9.9)	9 (6.9)
Unknown	143 (13.0)	175 (21.3)	72 (12.7)	19 (14.5)
Pancreas reconstruction				
PJ	963 (87.9)	809 (98.3)	560 (99.1)	129 (98.5)
PG	5 (0.4)	3 (0.4)	2 (0.4)	0 (0.0)
None	128 (11.7)	11 (1.3)	3 (0.5)	2 (1.5)
Vascular resection				
No	830 (75.7)	784 (95.3)	562 (99.5)	128 (97.7)
Yes	266 (24.3)	39 (4.7)	3 (0.5)	3 (2.3)
Combined operation				
No	964 (88.0)	801 (97.3)	545 (96.5)	112 (85.5)
Yes	132 (12.0)	22 (2.7)	20 (3.5)	19 (14.5)
Operation duration (minutes)	341.0 ± 70.4	332.1 ± 67.2	302.4 ± 57.7	330.0 ± 91.5
EBL (mL)	581.7 ± 630.0	551.0 ± 528.0	437.4 ± 477.3	478.6 ± 353.0
Intraoperative transfusion				
No	918 (83.8)	680 (82.6)	517 (91.5)	103 (78.6)
Yes	178 (16.2)	143 (17.4)	48 (8.5)	28 (21.4)

BMI: body mass index, DM: diabetes mellitus, ASA: American Society of Anesthesiologists, CEA: carcinoembryonic antigen, CA19-9: carbohydrate antigen 19-9, PPPD: pylorus preserving pancreaticoduodenectomy, PRPD: pylorus resecting pancreaticoduodenectomy, HPD: hepatopancreaticoduodenectomy, TP: total pancreatectomy, PJ: pancreaticojejunostomy, PG: pancreaticogastrostomy, EBL: estimated blood loss. Combined operation: splenectomy (86 cases), colectomy (32 cases), appendectomy (18 cases), gastrectomy (9 cases), and others.

**Table 3:** Pathologic and postoperative features of 2615 patients with malignant tumor (Mean ± Standard deviation and number (percent)).

	Pancreas (*n* = 1096)		Bile Duct (*n* = 823)		Ampulla (*n* = 565)		Duodenum (*n* = 131)	
Pathology								
Adenocarcinoma	1003 (91.5)		811 (98.5)		531 (94.0)		105 (80.1)	
Endocrine carcinoma	29 (2.7)		2 (0.2)		11 (1.9)		10 (7.6)	
Mucinous carcinoma	31 (2.8)		0 (0.0)		14 (2.5)		4 (3.1)	
Signet ring cell carcinoma	0 (0.0)		4 (0.5)		4 (0.7)		3 (2.3)	
Acinar cell carcinoma	4 (0.4)		0 (0.0)		0 (0.0)		0 (0.0)	
Metastatic cancer	22 (2.0)		3 (0.4)		0 (0.0)		4 (3.1)	
Etc.	7 (0.6)		3 (0.4)		5 (0.9)		5 (3.8)	
Differentiation								
Well	92 (8.4)		108 (13.1)		164 (29.0)		27 (20.6)	
Moderately	597 (54.5)		457 (55.5)		265 (46.9)		60 (45.8)	
Poorly	264 (24.1)		209 (25.4)		105 (18.6)		24 (18.3)	
Undifferentiated	23 (2.1)		11 (1.4)		5 (0.9)		4 (3.1)	
Unknown	120 (10.9)		38 (4.6)		26 (4.6)		16 (12.2)	
Tumor size (cm)	3.1 ± 1.7		2.8 ± 1.4		2.1 ± 1.3		4.4 ± 2.9	
T stage	Tis	2 (0.2)	T1	169 (37.8)	Tis	8 (1.4)	Tis	2 (1.5)
	T1a	5 (0.5)	T2	225 (50.3)	T1a	132 (23.4)	T1a	5 (3.8)
	T1b	7 (0.6)	T3	42 (9.4)	T1b	106 (18.8)	T1b	5 (3.8)
	T1c	191 (17.4)	T4	9 (2.0)	T2	121 (21.4)	T2	11 (8.4)
	T2	715 (65.2)	Metastatic	2 (0.5)	T3a	113 (20.0)	T3	23 (17.6)
	T3	153 (14.0)	Unknown	376	T3b	85 (15.0)	T4	81 (61.8)
	T4	1 (0.1)			T4	0 (0.0)	Metastatic	4 (3.1)
	Metastatic	22 (2.0)						
N stage								
N0	426 (38.9)		542 (65.8)		366 (64.8)		42 (32.1)	
N1	439 (40.0)		217 (26.4)		142 (25.1)		43 (32.8)	
N2	231 (21.1)		64 (7.8)		57 (10.1)		46 (35.1)	
Harvested LN	22.7 ± 13.0		20.5 ± 10.6		20.4 ± 10.5		21.2 ± 12.8	
Metastatic LN	2.2 ± 3.4		1.0 ± 2.1		1.1 ± 2.6		2.7 ± 3.4	
M stage								
M0	1081 (98.6)		821 (99.8)		563 (99.6)		128 (97.7)	
M1	15 (1.4)		2 (0.2)		2 (0.4)		3 (2.3)	
Resection margin								
R0	804 (73.4)		760 (92.3)		559 (98.9)		123 (93.8)	
R1	271 (24.7)		58 (7.1)		4 (0.7)		4 (3.1)	
R2	21 (1.9)		5 (0.6)		2 (0.4)		4 (3.1)	
Lymphovascular invasion								
No	211 (19.2)		231 (28.1)		181 (32.0)		13 (9.9)	
Yes	450 (41.1)		210 (25.5)		204 (36.1)		40 (30.5)	
Unknown	435 (39.7)		382 (46.4)		180 (31.9)		78 (59.6)	
Perineural invasion								
No	68 (6.2)		124 (15.1)		267 (47.2)		20 (15.3)	
Yes	802 (73.2)		535 (65.0)		101 (17.9)		31 (23.6)	
Unknown	226 (20.6)		164 (19.9)		197 (34.9)		80 (61.1)	
Length of stay (days)	15.1 ± 11.5		17.9 ± 13.1		15.2 ± 9.3		15.6 ± 11.5	
POPF								
No (Biochemical leak)	927 (95.7)		719 (88.4)		506 (90.0)		115 (89.1)	
Grade B	34 (3.5)		80 (9.9)		46 (8.2)		12 (9.3)	
Grade C	8 (0.8)		14 (1.7)		10 (1.8)		2 (1.6)	
TP	127		10		3		2	
Clavien–Dindo classification								
No complication	684 (62.4)		438 (53.2)		324 (57.4)		71 (54.2)	
I	62 (5.7)		48 (5.8)		29 (5.1)		8 (6.1)	
II	169 (15.4)		137 (16.7)		99 (17.5)		28 (21.3)	
IIIa	121 (11.0)		138 (16.8)		84 (14.9)		19 (14.5)	
IIIb	19 (1.7)		24 (2.9)		13 (2.3)		1 (0.8)	
IVa	24 (2.2)		25 (3.0)		12 (2.1)		1 (0.8)	
IVb	4 (0.4)		2 (0.3)		3 (0.5)		1 (0.8)	
V	13 (1.2)		11 (1.3)		1 (0.2)		2 (1.5)	

LN: lymph node, POPF: postoperative pancreatic fistula, TP: total pancreatectomy.

**Table 4:** Chronologic changes of postoperative features of all patients (Mean ± Standard deviation and number (percent)).

	*n* = 2903 Except TP				
	1994–1998 (*n* = 61)	1999–2003 (*n* = 223)	2004–2008 (*n* = 549)	2009–2013 (*n* = 958)	2014–2018 (*n* = 1112)
POPF					
No (Biochemical leak)	60 (98.4)	220 (98.7)	499 (90.9)	856 (89.4)	1021 (91.8)
Grade B	1 (1.6)	3 (1.3)	40 (7.3)	91 (9.5)	76 (6.8)
Grade C	0 (0.0)	0 (0.0)	10 (1.8)	11 (1.1)	15 (1.3)
	**Total *n* = 3078**				
	**1994–1998** **(*n* = 79)**	**1999–2003** **(*n* = 251)**	**2004–2008** **(*n* = 581)**	**2009–2013** **(*n* = 1013)**	**2014–2018** **(*n* = 1154)**
Length of stay (days)	28.0 ± 14.0	22.8 ± 15.9	17.9 ± 14.0	14.4 ± 8.4	13.8 ± 9.5
Clavien–Dindo classification					
No complication	61 (77.2)	210 (83.7)	324 (55.8)	570 (56.3)	654 (56.7)
I	10 (12.7)	15 (6.0)	31 (5.3)	86 (8.5)	28 (2.4)
II	3 (3.8)	6 (2.4)	123 (21.2)	168 (16.6)	208 (18.0)
IIIa	3 (3.8)	8 (3.2)	76 (13.1)	130 (12.8)	188 (16.3)
IIIb	2 (2.5)	10 (4.0)	15 (2.6)	18 (1.8)	22 (1.9)
IVa	0 (0.0)	0 (0.0)	7 (1.2)	27 (2.7)	35 (3.0)
IVb	0 (0.0)	0 (0.0)	1 (0.2)	3 (0.3)	8 (0.7)
V	0 (0.0)	2 (0.8)	4 (0.7)	11 (1.1)	11 (1.0)

TP: total pancreatectomy, POPF: postoperative pancreatic fistula.

**Table 5:** Chronologic differences of survival rates for those with each adenocarcinoma from 1994 to 2016.

	Total No.	Median Survival	1 Year Survival Rate	3 Year Survival Rate	5 Year Survival Rate
Pancreatic Adenocarcinoma					
1994–2004 (11 years)	64	19.3 months	39%	20%	14%
2005–2010 (6years)	269	21.0 months	44%	30%	26%
2011–2016 (6 years)	477	29.5 months	55%	38%	34%
Bile duct Adenocarcinoma					
1994–2004 (11 years)	164	36.8 months	60%	41%	35%
2005–2010 (6 years)	214	44.0 months	66%	48%	40%
2011–2016 (6 years)	313	96.0 months	78%	69%	63%
Ampullary Adenocarcinoma					
1994–2004 (11 years)	69	102.0 months	74%	62%	54%
2005–2010 (6 years)	149	120.0 months	78%	64%	62%
2011–2016 (6 years)	231	96.0 months	84%	75%	72%
Duodenal Adenocarcinoma					
1994–2004 (11 years)	5	21.0 months	40%	0%	0%
2005–2010 (6 years)	41	46.5 months	68%	49%	44%
2011–2016 (6 years)	39	96.0 months	64%	61%	61%
